# Does drug compliance change in asthmatic patients during pregnancy?

**DOI:** 10.1186/2049-6958-8-38

**Published:** 2013-06-11

**Authors:** Insu Yilmaz, Ferda Oner Erkekol, Sevki Celen, Mujdegul Zayifoglu Karaca, Omur Aydin, Gulfem Celik, Zeynep Misirligil, Dilsad Mungan

**Affiliations:** 1 Department of Chest Diseases, Division of Immunology and Allergy, Ankara University, School of Medicine, Dikimevi, Ankara 06100, Turkey; 2 Atatürk Chest Diseases & Thoracic Surgery Training and ResearchHospital, Unit of Allergy, Ankara, Turkey; 3 Department of Obstetrics and Gynecology, Zekai TahirBurak Women’s Health Education and Research Hospital, Ankara, Turkey

**Keywords:** Asthma, Drug compliance, Pregnancy, Uncontrolled asthma

## Abstract

**Background:**

Pregnant women with asthma are recommended to maintain optimal therapeutic management during pregnancy. Uncontrolled, symptomatic asthma may increase the risk of adverse peri-natal outcomes; thus adequate regular anti-asthmatic treatment must be given to provide optimal asthma control during pregnancy. However, doubts about the safety of asthmatic drugs can affect pregnant asthmatic patients’ drug compliance. The aim of this study was to assess behavioral differences in drug compliance among pregnant asthmatic patients.

**Methods:**

Thirty two asthmatic and 121 healthy pregnant women were enrolled in the study. Structured face-to-face interviews were conducted after delivery. The interviews included disease characteristics, drug compliance and patients’ own perspective for asthma status prior to and during pregnancy. In addition, medical and pregnancy history, pregnancy complications and outcomes, and newborn characteristics were recorded.

**Results:**

In our study group the rates of hospitalization, emergency room visits and systemic steroid use in the year before pregnancy were 13%, 46.9% and 18.8%, respectively. The rate of regular asthma medication use was only 32% at that period and increased to 44% during pregnancy. However, hospitalization, emergency room visits, systemic steroid usage rates remained unchanged and according to patients’ own evaluations, 44% of asthmatics pointed out that their asthma had worsened during pregnancy. No statistically significant difference was detected in terms of pregnancy/labour complication between asthmatic and non-asthmatics.

**Conclusions:**

Contrary to some previous studies, in our study regular use of asthma drugs increased during pregnancy. The uncontrolled condition of their asthma before and during pregnancy and the idea that their asthma worsened during pregnancy might force the patients to use medication more regularly.

## Background

Asthma is one of the most common chronic medical conditions, accounting for complications in up to 8% of pregnant women [1]. Women with well-controlled asthma during pregnancy have been reported to have healthy pregnancy periods with excellent maternal and peri-natal outcomes. Treatment strategies do not differ between pregnant and non-pregnant asthmatic women and include the medications given for the maintenance and reliever therapy and for the treatment of asthma attacks as well [2]. However, asthma treatment during pregnancy has led to discussion concerning medications and complications of pregnancy. These concerns usually lead to a behavioral change in pregnant asthmatics against the medications that they had previously used. Results from research seem to corroborate this. One study reported that doubts about safety and possible teratogenicity of medications lead 40% of pregnant women to reduce or cease asthma medications, particularly in the first trimester, which results in an increase in the consumption of rescue medication, an increased number of exacerbations, and reduced asthma control in pregnant asthmatics with moderate to severe disease [2]. Similarly, in another study, 39% of asthmatic women either discontinued their asthma medications or reduced their dosage during pregnancy [3]. The authors also stated that 82% of women using inhaled corticosteroids (ICS) had concerns about the possibility of birth defects occurring if they became pregnant [3]. Furthermore, it was reported in another study that women with asthma significantly decrease their asthma medication use from five to 13 weeks of pregnancy. During the first trimester, there was a 23% decline in ICS prescriptions, a 13% decline in short-acting beta-agonist (SABA) prescriptions, and a 54% decline in rescue corticosteroid prescriptions [4].

It is well known that there are regional and cultural differences in the control of asthma and drug compliance [5]. In this study, we sought to observe the behavior of pregnant women with asthma regarding drug usage in a real life setting in our country. To the best of our knowledge, to date there have been no studies in our population examining how drug compliance changes in asthmatic pregnant patients. Therefore, the aim of this study was to assess behavioral differences regarding drug compliance among pregnant women.

## Methods

### Study population

One hundred and fifty-three pregnant women admitted to a Women’s Health Training and Research Hospital in their third trimester were included in this cross-sectional study. Subjects were randomly selected at the Department of Obstetrics and Gynecology. Four consecutive non-asthmatic pregnant women were included for each asthmatic pregnant woman. All women had been admitted to the hospital for delivering. No asthmatic pregnant had been followed for asthma management by the chest physicians in the study during her pregnancy. Asthmatic pregnants were selected according to the presence of a history of physician-diagnosed asthma, a physician report confirming asthma diagnosis and use of asthma medication for a period of at least 1 year before pregnancy. Following this evaluation, the patients were allocated into two groups; group I consisted of asthmatic pregnants (n = 32), and group II was comprised of non-asthmatic pregnants (n = 121). Structured face-to-face interviews were conducted via a questionnaire. The interviews were performed by a physician within 24 hours of delivery, and the patients’ demographics and disease characteristics, and asthma medication use before and during pregnancy were evaluated by the same physician. Also the number of hospitalizations, admission to an emergency department, use of oral steroids because of asthma, asthma medications, regular drug use, and the patients’ views on their asthma status before and during pregnancy were recorded. In this study, asthma status was only evaluated from patients’ perspective. In the questionnaire, they were asked to judge their asthma status before and during pregnancy and they made a decision whether it got worse or improved or stayed stable. Like other parameters listed above, regular drug use was also determined according to patients’ declarations. Patients were asked if they had been using ICS regularly in the previous year before pregnancy and during pregnancy. The study was approved by the local ethics committee. All patients gave verbal informed consent to be included in the study.

### Parameters

Gestational age was defined as the duration of the pregnancy in days, and preterm delivery as birth occurring before 37 completed weeks of gestation, according to the classification system of the World Health Organization. Small for gestational age (SGA) and low birth weight (LBW) were defined as a birth weight below the 10% percentile for each gender by gestational age (completed weeks) and as a birth weight below 2,500 g, respectively. The assessment of LBW was restricted to deliveries after the 37^th^ week of gestation. The Apgar score cut-off value was 8 since adverse effects are associated with Apgar scores < 8.

### Statistical analysis

All analyses were performed using computer software (SPSS version10:0; SPSS; Chicago, IL). Nominal values are described as percentages. Descriptive statistics are expressed as mean ± SD. Categorical data were tested using the chi-square statistics. Mann–Whitney U test was used for comparison of the continuous variables. A P less than 0.05 was considered statistically significant.

## Results

The two groups were statistically different in terms of age and profession. Asthmatic pregnant women were older, and in the asthmatic group there were more housewives than in non-asthmatic one (Table 1).

**Table 1:** Demographic features and pregnancy history of subjects

	**Pregnants with asthma n = 32**	**Pregnants without asthma n = 121**
**Age, years (mean±SEM)**	28.7±6.4	25.9±5.3*
**Profession (housewife) n (%)**	31 (96.9%)	100 (82.6%)*
**Educational status (primary school) n (%)**	21 (65.6%)	72 (59.5%)
**Smoking during pregnancy n (%)**	4 (12.5%)	18 (14.9%)
**Duration of asthma, years median (min-max)**	5 (1–20)	-
**Gravity, median (min-max)**	2 (1-7)	2 (1-7)
**Parity, median (min-max)**	2 (0–5)	1 (1-5)
**Abortus, median (min-max)**	0 (0–2)	0 (0–2)

*p<0.05.

Seven asthmatic women did not use any medication for their asthma before and during pregnancy. While the rate of regular asthma medication use was 32% (n = 8) before pregnancy and 44% (n = 11) during pregnancy term, the rate of irregular asthma medication use was 68% (n = 17) before pregnancy and 56% (n = 14) during pregnancy term (p = 0.561). An increase of 12% was detected in regular use of asthma medication, and this was not statistically significant. In the group of asthmatic pregnants affirming that they had been taking their asthma drugs regularly before pregnancy, 87.5% (n = 7) kept using their regular drug and attended regular follow up for asthma during pregnancy. 23.5% (n = 4) of asthmatic pregnants who used asthma medications irregularly before pregnancy stated that they began to use asthmatic drugs regularly during pregnancy.

Among asthmatic pregnant women, 52% (n = 13) used inhaled anti-inflammatory agents, 40% (n = 10) used SABA (at least two times a week), and 40% (n = 10) used long-acting beta2 agonists (LABA) (two subjects used only LABA, eight subjects used combination of ICS+LABA) during pregnancy. The usage rates of asthma drugs according to the medication class were not significantly different between the periods before and after pregnancy in asthmatic subjects. In this study the ICS and LABA drugs used by patients were fluticasone or budesonide and formeterol or salmeterol, respectively.

The rates of hospitalizations 13% (n = 4), emergency visits 46.9% (n = 15), and systemic steroid use 18.8% (n = 6) recorded in the previous year remained unchanged during the pregnancy term [13% (n = 4), 46.9% (n = 15), and 21.9% (n = 7), respectively].

According to the patients’ own evaluations, asthma status improved in 28% of pregnant patients, remained unchanged in 28%, and worsened in 44% of the subjects during pregnancy.

There was no difference in terms of pregnancy/labour complication, Apgar score, offspring malformation between asthmatic and non-asthmatic pregnants (Table 2).

**Table 2:** Obstetric variables in pregnant women with and without asthma

	**With asthma**	**Without asthma**	**p**
**Labour complication**	9.4%	14.9%	> 0.05
**Pregnancy complication**	28.1%	19.8%	> 0.05
**Score of APGAR**	100%	97.5%	>0.05
**Mean birth weight (gr)**	3315	3245	>0.05
**Mean birth week**	38.5	38.6	>0.05
**Malformation**	0%	2.5%	>0.05

## Discussion

Our results revealed that almost half of the pregnant women with asthma used their controller drugs regularly during pregnancy. Furthermore, the regular use of asthma drugs increased during pregnancy when compared to the pre-pregnant period. However, some clinical parameters, such as hospital and emergency room admissions and the use of oral steroids due to asthma, showed that asthma was not controlled well enough in many asthmatic pregnants during pregnancy.

One of the findings of our study was that asthma medication use did not change in asthmatic pregnants, whereas an increase of 12% was detected in regular asthma medication use although it is not statistically significant. This result is not consistent with previous studies, which report a decrease in drug usage among asthmatic pregnants [3,4]. In one study [3], 39% of women were reported to discontinue or reduce asthma medication use during pregnancy, mostly without consulting with their doctor, mainly because of concerns related to the safety of these drugs during pregnancy. The most significant decrease in asthma medication use in early pregnancy was relative to inhaled anti-inflammatory agents and rescue corticosteroids, with a 23% and 54% decrease, respectively. In the second trimester there was a rebound in the use of inhaled and rescue corticosteroids, although their use remained lower than that of pre-pregnancy [4]. The inconsistency of our findings with previous literature may be related to some of the features of the group in our study. Asthma control seems to be poor in approximately half of asthmatic pregnant women before and during pregnancy according to the rates of hospitalizations, emergency service visits, and oral steroid usage. Consequently, they needed to use medications regularly to keep their asthma in a more stable state. Considering these evaluations, we can infer that poor asthma control during pregnancy may positively influence pregnant women to use their asthma medications.

It is widely accepted that treatment with asthma medications is a safer option for pregnant women with asthma than having asthma symptoms and exacerbations [6]. Inhaled corticosteroids have been advocated as the first line therapy for patients with asthma during pregnancy. There are several drugs that can be added to ICS, i.e. short and long acting beta agonist bronchodilators, which may be elected according to the severity of bronchial constriction and the frequency of crises. The use of inhaled SABA, as required, is currently recommended for all levels of asthma in pregnant women. However, there is insufficient data currently available regarding the safety of LABA during pregnancy although safety is considered likely based on the inhalational route and the generally reassuring data for SABA [7]. Any treatment for acute asthma during pregnancy should follow recommended guidelines since uncontrolled asthma increases the risk of pregnancy complications (low birth weight and premature delivery) more than the eventual medication risks to pregnancy [8-12]. In the present study a fairly high proportion (40%) of pregnant women with asthma used LABA, and this may reflect that they have moderate to severe asthma and LABA were given to achieve the control of the disease.

In this analysis, interviews with the patients were performed within 24 hours from delivery. The patients were asked about asthma related items, such as use of asthma medications, number of hospitalizations, and emergency department admissions. Although it looks like a limitation in terms of over/under reporting of the questioned items retrospectively, this evaluation enabled us to collect information under real life conditions. If the patients had been included in the study at the beginning of their pregnancy, they may have behaved more compliantly, and the results of the study would not reflect reality.

There are many limitations of this study. Since this is a cross sectional, questionnaire based study, all data used in this study (like asthma diagnosis, regular drug usage, hospitalization, emergency visit, systemic steroid usage rates) depend on the patients’ statements. None of the patients had been followed by the chest physicians in the study before or during pregnancy for their asthma management, thus we did not have chance to know their management strategy and to check the accuracy of their statements. And again since we did not follow up information, asthma control status of the patients during pregnancy was not clear. It was only evaluated from patients’ perspective and we tried to put forward an idea about asthma control of the patients depending on the rates of hospitalization, emergency visit, and systemic steroid usage. However, we believe that if the patients had been followed by a physician of this study during pregnancy, they would have reported data not consistent with their usual behavior. Another important limitation of this study is the small number of cases. Although the small number of subjects limits us to make firm conclusions, this first study gives information on drug usage in asthmatic pregnant women in our country.

## Conclusion

In conclusion, regular use of asthma drugs increased during pregnancy in our study. The uncontrolled condition of asthma before and during pregnancy and the idea that asthma worsened during pregnancy might force the patients to use medication more regularly. Therefore, particular attention should be paid to the education of physicians and asthmatic women, concerning asthma control before and during pregnancy.

## Availability of supporting data

The data sets supporting the results of this article are included within the article.

## Competing interest

Authors declare that they have no competing interests.

## Authors’ contributions

IY Study design, inclusion of patients, data entrance, writing manuscript. FOE Study design, inclusion of patients, statistical analysis, writing manuscript. SC, MZK, OA, GC and ZM: Study design, inclusion of patients, significant contribution to written manuscript. DM Study design, inclusion of patients, writing manuscript. All authors read and approved the final manuscript.
